# The history of AIDS exceptionalism

**DOI:** 10.1186/1758-2652-13-47

**Published:** 2010-12-03

**Authors:** Julia H Smith, Alan Whiteside

**Affiliations:** 1 Peace Studies Department, University of Bradford, Bradford, West Yorkshire, UK; 2 Health Economics and HIV/AIDS Research Division, University of KwaZulu-Natal, Westville Campus, Durban, South Africa

## Abstract

In the history of public health, HIV/AIDS is unique; it has widespread and long-lasting demographic, social, economic and political impacts. The global response has been unprecedented. AIDS exceptionalism - the idea that the disease requires a response above and beyond "normal" health interventions - began as a Western response to the originally terrifying and lethal nature of the virus. More recently, AIDS exceptionalism came to refer to the disease-specific global response and the resources dedicated to addressing the epidemic. There has been a backlash against this exceptionalism, with critics claiming that HIV/AIDS receives a disproportionate amount of international aid and health funding.

This paper situations this debate in historical perspective. By reviewing histories of the disease, policy developments and funding patterns, it charts how the meaning of AIDS exceptionalism has shifted over three decades. It argues that while the connotation of the term has changed, the epidemic has maintained its course, and therefore some of the justifications for exceptionalism remain.

## Background

In the 30 years since it was first recognized, HIV has spread globally. An estimated 25 million people have died, and about 33 million people are currently living with HIV/AIDS. The epidemic is not homogenous; the global picture is diverse. In wealthy countries, most of Latin America, Asia, north Africa and the Middle East, infections are concentrated in particular geographical locations and among specific population groups. These are often socially and politically marginalized populations, including injecting drug users, men who have sex with men, and commercial sex workers.

Generalized epidemics are found in eastern, central and southern Africa, where between 5% and 30% of adults are infected. However, even here, specific groups, such as women, remain disproportionately at risk. The development of HIV "risk environments" [1] has been shaped by social-structural, economic and political factors specific to each context, and indicated by differing prevalence rates.

In the history of public health, HIV/AIDS is unique in terms of how it is spread and attacks the body and because of its widespread and long-lasting demographic, social, economic and political impacts. HIV/AIDS is a long-wave event: an epidemic that, where it is most prevalent, will have consequences that will be felt for generations [2]. Just as the epidemic is distinctive, so has been the response, though for opposite reasons. Despite the progression of the epidemic, the HIV/AIDS response has been characterized by both lack of action and fevered aid at different points in time. This disease-specific response has become known as AIDS exceptionalism. The word, "exceptionalism", means to treat or to give something the status of being exceptional, and can be positive or negative.

AIDS exceptionalism began as a Western response to the originally terrifying and lethal nature of the virus, which disproportionately affected specific groups. The first activists argued that HIV/AIDS required an exceptional response in order to protect the rights of those infected, to generate resources to assist them and to curb a then mysterious epidemic. More recently, AIDS exceptionalism came to refer to the disease-specific global response. This international response was unprecedented, as the commitment of resources exceeded any other health cause.

International organizations, such as the Joint United Nations Programme on HIV/AIDS (UNAIDS), the Global Fund to Fight AIDS, Tuberculosis and Malaria (the Global Fund) and the US President's Emergency Plan for AIDS Relief (PEPFAR), were formed to specifically address HIV/AIDS. In the past few years, there has been a backlash against this exceptionalism, with critics claiming that HIV/AIDS receives a disproportionate amount of international aid and health funding, and that this has implications for other health issues.

This paper reviews the histories of the disease, policy developments and funding patterns to chart how the meaning of AIDS exceptionalism has shifted over three decades. It argues that, while the connotation of the term has changed, the epidemic has maintained its course, and therefore some of the justifications for exceptionalism remain.

## Discussion

### The rise of exceptionalism in the West

In the early 1980s, when previously healthy, mostly homosexual young men began dying, the unknown cause and rising numbers of deaths combined with homophobia to generate a response of blame and fear. Extreme religious right-wing advocates spoke of divine punishment for "sinful" lifestyles [3]. The disease was initially termed the gay-related immunodeficiency disease (GRID), or "the gay plague" [4]. Homosexual men were openly discriminated against when they tried to access health services.

As haemophiliacs, women and children in the West increasingly presented with the same symptoms, it became clear that the cause of illness was not related to sexual orientation. In 1983, the human immunodeficiency virus (HIV) was identified as the cause of AIDS. The realization that HIV could spread to the general public, and that it was linked to the life-giving and pleasurable acts of sexual intercourse, resulted in increased hysteria. Governments, the media and scientists sought a quick response: "A sense of urgency defined the problem, and the public information materials developed in this period often emphasized danger at the expense of clear information about prevention measures" [5]. Even as information about modes of transmission (unprotected sexual intercourse, injecting drug use, and from mother to infant) became more accurate, the original fear and stigma prevailed.

The gay rights movement, building on the momentum it had gained in the preceding decades, began campaigning for HIV/AIDS to be viewed as a human rights issue. Advocates argued that infection was not the only risk; if found positive, individuals also faced harmful discrimination [6]. In this, they were supported by public health officials, who feared that stigma would prevent those at risk from getting tested, and those infected from accessing health services:

Avoiding compulsory measures such as isolation and quarantine, which were so much a part of the public health tradition, was all the more crucial, since the people with increased risk -- gay and bisexual men, drug users, and their sexual partners -- were already socially vulnerable ... Policies and practices that appeared to threaten such persons could only drive the epidemic underground and make it more difficult to work with the populations within which HIV was spreading [7].

Recognizing the unique needs of populations at risk of HIV infection, an exceptionalist alliance, including the gay community, liberal and left-wing parties, and the healthcare and psychosocial professions, was formed to advocate for a unique response. Bayer writes, "The embrace of exceptionalism must be understood in broad political terms, as representing in large measure, a singular victory on the part of gay men, their community-based organisations and their allies" [8]. The alliance promoted the empowerment of those groups most at risk, and the assurance that their rights would be protected.

During the 1980s, public health adopted a human rights framework that took societal-based vulnerability into consideration and increasingly became involved in societal transformation efforts [5]. HIV/AIDS was positioned as not only a health condition, but also as a social issue that required a political, as well as a medical, response [4]. The scientific establishment's control on public health was challenged, and a new type of public health initiative was called for: one that provided counselling, protected privacy, and empowered the patient. Lazzarini summarizes:

Descriptively, exceptionalism posited that in the early years of the HIV epidemic, HIV was considered so different, so "exceptional" in comparison to other communicable diseases that advocates and public health officials agreed that HIV policy should cater to the uniqueness of the epidemic rather than treat it like all other communicable diseases. Supposedly, the argument goes, public fear was so great, the political power of gay men so substantial, and concern over stigmatization so real, that public health authorities abandoned "traditional" approaches to communicable disease control in favor of a civil liberties approach [9].

This public health approach helped contain the epidemic among those groups most at risk, and meet, to varying degrees, their specific needs. New infections in the United States fell from approximately 130,000 in 1984 to about 60,000 in 1991 [10]. The feared general epidemic never occurred in the West.

The hysteria surrounding HIV/AIDS faded; the media and public policy lost interest. The human rights approach that had previously been revolutionary was integrated into existing public health traditions that included education, technical solutions and regular testing. In 1991, Bayer wrote, "As AIDS has become less threatening, the claims of those who argued that the exceptional threat would require exceptional policies have begun to lose their force" [11]. Highly affected communities continued to advocate for the rights of people living with HIV and AIDS (PLHIV) and against stigma, but much of the sense of urgency among the general public was lost.

When antiretroviral treatment (ART) was unveiled at the 1996 International AIDS Conference in Vancouver, Canada, AIDS was transformed into a treatable disease. The advent of treatment shifted Western priorities of response: "The availability of more advanced antiretroviral therapies has made it possible to treat effectively those with HIV infection, thereby increasing the importance of early identification and tracking. These developments establish a strong case for moving beyond HIV exceptionalism and treating HIV antibody tests like other blood tests" [12].

As technical solutions of testing and treatment gained priority, the social movement that had spurred the early HIV/AIDS response continued to fade from public awareness. This shift was assisted by a rapid fall in the price of the drugs. Had they remained expensive, AIDS exceptionalism would have been perpetuated. As Casaratt and Lantos noted, "Medical therapy has become more effective but also prohibitively expensive. A medical tragedy has been transformed into a financial crisis and society has responded by establishing special programs and sources of funding for AIDS. These manoeuvres parallel earlier approaches to HIV testing and reporting that have collectively come to be known as exceptionalism" [13]. The mobilization of resources to make treatment available in the West altered HIV/AIDS from a lethal disease to a manageable chronic illness. By 2000, AIDS exceptionalism, as it had originally been conceived, was over.

Throughout the rise and fall of Western AIDS exceptionalism, the growing global epidemic remained largely ignored by the international community [14]. During the 1980s, reports from Africa of similar diseases and symptoms were ignored [15]. There were few attempts, and even resistance, to linking HIV/AIDS to "slim disease", as it was called in central Africa [14]. When the African AIDS epidemic was finally recognized, in the late 1980s, little international attention or resources were forthcoming. In 1990 and 1991, only 6% of the total global spending for HIV prevention went to the developing world [16]. For international organizations, after the first fears of a Western rampant unstoppable epidemic were allayed, HIV/AIDS was not a priority.

The Global Programme on AIDS in the World Health Organization (WHO) lacked both the funding and capacity to respond to the epidemic. Outside of WHO, HIV/AIDS was not on the agenda of other United Nations (UN) agencies. International responses between 1986 and 1996 were characterized by denial, underestimation and over-simplification [17]. AIDS exceptionalism was originally a Western-focused phenomenon that advocated for the rights of those most affected and became a decreasing public force as effective treatment was rolled out in North America and Europe.

### International exceptionalism

In 1996, UNAIDS was formed as a joint programme of UN agencies engaged in the AIDS response [17]. In 1998, it published its first set of comprehensive data on HIV/AIDS, demonstrating the global scale of the epidemic, and increasing awareness about the generalized epidemic in parts of sub-Saharan Africa. Such data provided an information arsenal for those advocating for increased resources for HIV/AIDS in mid- and high-prevalence developing countries [17]. UNAIDS, adopting public health rhetoric from the early AIDS response in the West, stressed the need for comprehensive responses that reached beyond a medical approach, and began advocating for increased funding for AIDS programmes [18]. During the same period, donor countries began to scale up international aid contributions. General overseas development assistance increased from US$53.6 billion in 2000 to US$61.1 billion in 2003 [19]. This aid was targeted towards issues perceived as "global", such as poverty, debt relief and communicable diseases.

HIV/AIDS gained prominence among these issues through the language of securitization and globalization. In 2000, US Vice President Al Gore said, "It [HIV] threatens not just individual citizens, but the very institutions that define and defend the character of a society ... It strikes at the military, and subverts the forces of order and peacekeeping" [20]. The US National Intelligence Council then produced *The Global Infectious Disease Threat and Its Implications for the United States *[21]. Six months later, the UN Security Council passed Resolution 1308, stating, "The HIV/AIDS pandemic, if unchecked, may pose a risk to stability and security" [22]. In 2001, UN Secretary General Kofi Annan called for a "war chest" of $7-10 billion to address the global HIV/AIDS crisis [23].

The Pretoria-based Institute for Security Studies wrote, "The severe social and economic impact of HIV/AIDS, and the infiltration of the epidemic into the ruling political and military elites and middle classes of developing countries may intensify the struggle for political power to control scarce state resources. Such dynamics, even singularly, have the potential to lead to political instability" [24].

It was argued that HIV/AIDS could hinder processes of democratization by undermining social development and intensifying the struggle for resources. It was further suggested that AIDS orphans could contribute to social unrest as they made likely recruits for terror and rebel groups: "Bluntly put, those who are orphaned may be indifferent to prevailing norms and values, may look for salvation to millenarian and fundamentalist beliefs of one kind or another, and may ultimately do this with assistance from a Kalashnikov or a bomb" [2].

While there was little evidence to actually link HIV/AIDS prevalence and security issues [25], the discourse of global threats drew international attention to the epidemic. Barnett and Prins write, "The combination of AIDS, orphans and terror begins to take on an independent life, perhaps regardless of either the strength of the evidence or the precise value of the parallel" [25]. HIV was seen as a virus that could have widespread repercussions for the most affluent and powerful, even though risk of infection and disease spread in these populations had abated. The epidemic was positioned as a homogenous issue with impacts for both the developed and developing world.

However, as policy and activists spoke of a global HIV/AIDS epidemic, it became apparent that, like many diseases that are expensive to treat, how this epidemic was experienced differed drastically by region. ART, available from 1996, proved effective in curbing AIDS deaths in those regions that could afford the high costs of medications (originally more than $25,000 per patient per year). In those countries with mid to high HIV/AIDS prevalence, ART remained unaffordable, even while costs dropped to about $10,000 per patient per year by 2000 and to less than $100 by 2010. A mid to high disease burden combined with lack of health resources in much of the developing world, making the benefits of ART beyond the reach of most domestic health budgets. While AIDS had become a chronic disease in the West, in most of the developing world. it was still a death sentence.

What made this discrepancy unique was the international mobilization that occurred around it, positioning the inequity of treatment access for people living with HIV and AIDS (PLHIV) as an international human rights cause. The International AIDS Conference in Durban in 2000 called for treatment to be rolled out in the developing world and for prices of ART be cut. Activists in South Africa demanded that their government provide universal access to ART, despite political resistance and denial. The governments of India and Brazil took on the World Trade Organization, arguing for compulsory licensing that would enable them to manufacture cheaper generic medications. Activists in the United States, largely led by the PLHIV organization, ActUp, began a sustained campaign against the US government's support for pharmaceutical companies' patent legislation [26].

Arguments that ART could not be provided in the developing world due to limited capacity and poverty were challenged by the successful implementation of such programmes by health organizations like Médecins Sans Frontières [27]. Researchers found that people living in poverty adhered to medication regimes just as consistently, if not more so, as those living in the developed world. Economists argued that the benefits of ART (keeping populations healthy for longer) outweighed the costs of treatment [28], adding further fuel to the human rights argument.

In 2002, Botswana implemented the first universal access programmes in sub-Sahara Africa. In 2003, the South African Government gave in to local and international pressure and announced its public treatment programme. The World Health Organization launched the "3 × 5" campaign, aiming to place 3 million people on treatment by 2005. In 2006, 111 countries committed to achieving universal access to prevention, treatment, care and support by 2010 [29].

The development of international AIDS programmes combined with a favourable political environment to create a new discourse of AIDS exceptionalism. At the 2001 UN General Assembly Special Session on AIDS, 189 nations agreed that HIV/AIDS was a national and international development issue of the highest priority [30]. This translated into increased international funding for HIV/AIDS programmes. In 2002, the Global Fund to Fight AIDS, Tuberculosis and Malaria was established. In 2003, US President George W Bush pledged $15 billion toward PEPFAR. In 2004, Kofi Annan prioritized HIV/AIDS, stating, "AIDS is a new type of global emergency--an unprecedented threat to human development" [30].

In 2004, at the Copenhagen Consensus, a policy think tank in Denmark, a panel of eight prominent economists ranked controlling HIV/AIDS as the number one economic priority in terms of a cost-benefit analysis in health and nutrition [14]. UNAIDS Executive Director Peter Poit encapsulated the exceptionalist point of view, saying, "This pandemic is exceptional because there is no plateau in sight, exceptional because of the severity and longevity of its impact, and exceptional because of the special challenges it poses to effective public action" [31]. The world had realized the size and impact of the AIDS epidemic, and was treating it as an emergency.

### The end of AIDS exceptionalism?

Arguments against exceptionalism began to gain attention in 2007. The amount of funding allocated to HIV/AIDS was called into question, as were the types of programmes being implemented. Three important books put forward new arguments. Chin and Pisani focused on the Asian experience, suggesting that scientists, UNAIDS and AIDS activists accept certain myths about HIV epidemiology to maintain the political profile of AIDS, and their own jobs [32]. Pisani spoke of resources for HIV/AIDS programmes disappearing down an "ideological drain" [33]. They suggested the epidemic was overstated, and money and resources were being deployed in situations where HIV would not spread anyway. Epstein's *The Invisible Cure *focused on the African epidemic, suggesting that one of the main drivers of the epidemic in hyper-epidemic countries was concurrent sexual partnering, but that such sensitive social issues had not been openly or adequately addressed, despite the resources mobilized for curbing the epidemic [34].

The strongest argument against AIDS exceptionalism has centred on the claim that responses undermined health systems in developing countries. In a series of opinion pieces in the *British Medical Journal*, Roger England argued that HIV/AIDS was not the "global catastrophe" claimed by "AIDS exceptionalists", and that donor aid for HIV/AIDS was disproportionate to the contribution of HIV/AIDS to the global disease burden [35]. England asserted that it would have been more cost effective to put the money into bed nets, immunization and dealing with childhood diseases. He accused UNAIDS of creating "the biggest vertical programme in history", which diverted human resources from the public sector, created additional reporting requirements and poorly coordinated donor activities for governments to cope with, and removed national control over spending priorities [36]. He wrote:

It is no longer heresy to point out that far too much is spent on HIV relative to other needs and that this is damaging health systems. Although HIV causes 3.7 percent of mortality, it receives 25 percent of international healthcare aid and a big chunk of domestic expenditure ... Until we put HIV in its place, countries will not get the delivery systems they need [36].

England and others' commentaries highlighted that many diseases and health issues (such as malaria, under-nutrition and respiratory disorders) resulted in more deaths than those related to AIDS in many parts of the world, but were receiving less funding. Whether or not this neglect was because of the prioritization of the AIDS response or due to other factors was hotly contested.

UNAIDS and WHO responded defensively, noting that those against AIDS exceptionalism attributed the UN agencies more power than they had to address the epidemic [37]. Research and policy looked to justify the role of HIV/AIDS programmes within broader health and development frameworks. Yu *et al *argued that HIV/AIDS funding actually increased access to other health resources in many underserved areas [38]. A 2007 report by the Institute of Medicine, charged with monitoring PEPFAR, suggested that the vertical HIV/AIDS programme could contribute to improving overall health outcomes [39]. These debates have been engaged with in more detail elsewhere [40]; here, they indicate that the exceptionalism that was attributed to HIV/AIDS as a global issue was called into question and, occasionally, outright attacked.

The emergence of the most recent exceptionalism debate is concurrent to a shift in donor country spending and priorities. Some donor countries have begun redirecting HIV/AIDS funding; in 2009, the UK Department for International Development reassigned a portion of its AIDS funds to maternal and child mortality programmes and health system strengthening [41]. Médecins Sans Frontières reports that The Netherlands cut its HIV/AIDS spending by $70 million [27].

As these changes take place, proposals are emerging to reorganize disease-specific funds in new ways. Sachs and Pronyk argue that the Global Fund should increase its mandate to include health systems, maternal and child mortality and neglected tropical diseases [42]. Similarly, Ooms *et al *argue for transforming the Global Fund from a disease-specific fund into a "Global Health Fund" [43]. HIV/AIDS is being positioned as one of many health issues requiring global resources. The current and potential impacts for HIV/AIDS programmes resulting from changes in funding patterns have been well discussed [27]; here, they represent a current shift away from prioritizing HIV/AIDS as a global issue.

### The current AIDS epidemic and response

While the debate on the global exceptionality of AIDS continues, HIV/AIDS will prevent many sub-Saharan African countries from achieving the Millennium Development Goals (MDGs) [44]. In high-prevalence countries (those with more than 10% of adults infected: Botswana, Lesotho, Malawi, Mozambique, Namibia, South Africa, Swaziland, Swaziland, Zambia and Zimbabwe), AIDS is the leading cause of death [27]. The epidemic has been found to decrease gross domestic product, create food security threats, and negatively impact human resources. The consequent orphaning has created new social costs for the state and for the household to bear; the number of orphans due to AIDS in sub-Saharan Africa increased from 6.5 million in 2001 to 11.6 million in 2007 [45]. AIDS is the leading cause of death for women worldwide [46], directly impacting MDG three (to promote gender equality and empower women) and goal five (to improve maternal health). The direct link between HIV/AIDS and development in Africa remains.

The number of new HIV infections each year remains unacceptably high, and models of effective prevention programmes are few and far between. In the endemic countries, mass behaviour-change campaigns, such as the ABC (Abstain, Be Faithful and Use Condoms) strategy, have failed to achieve their intended impacts. Advocates are stressing the need to address the systemic contributors to risk and vulnerability.

In South Africa, where one of the largest epidemics continues to unfold, Venter writes that prevention efforts are failing: "500 000 South Africans are infected each year, and there is no sign that this is letting up. In 8 to 10 years' time, as they enter the AIDS stage, these 500 000 South Africans will need ARVs, and this will continue forever until a prevention strategy that works is implemented" [47].

In Western countries, though the epidemic is concentrated in specific population groups, infection rates among these groups are stable (but not declining). El-Sadre *et al *write from the American perspective:

For the past decade, however, progress has been stalled. It had been anticipated that effective antiretroviral therapy, with its suppressive effect on viral replication, would reduce the overall rate of new infections, but this expectation has not been realized. More than half a million Americans became infected with HIV in the past decade, including about 56,000 in the past year [10].

The authors advocate for interventions that "address the socioeconomic milieu in which HIV transmission occurs" [10]. The social-political and human rights approaches that originally motivated for an exceptional response remain relevant to prevention, but are over-shadowed by technical solutions, such as testing and treatment.

Meanwhile, more than half of those in need still do not have access to treatment, and treatment is posing new challenges for sustainable funding. Most countries with mid to high prevalence cannot afford the cost of treatment without international aid; as aid is reduced or cancelled, treatment programmes are threatened, drug resistance develops and large numbers of PLHIV die. In countries like Uganda, patients are being turned away from treatment clinics due to lack of resources; 300,000 Ugandans in need of treatment are denied their right to health [48]. These numbers will continue to grow if both effective prevention and sustainably funded treatment programmes are not forthcoming.

The gap between resources required to implement HIV/AIDS programmes and those available has continued to grow over the past three years [49]. In 2009, UNAIDS and WHO predicted that the $25.1 million needed for HIV/AIDS programmes in 2010 would not be forthcoming [49]. The total amount of Global Fund grants recommended for funding in 2009 was 35% lower than in 2008 [27]. In 2010, US President Barrack Obama increased funding to PEPFAR only minimally, allocating less to the Global Fund than in previous years, and causing HIV/AIDS groups to express concern that the amount allocated would not be enough to reach the targeted number of PLHIV in need of treatment [50].

De Waal writes, "Normalization in the sense of adjusting reality to take account of the miseries of AIDS can be found in many places, when it is looked for. Even the statistics become numbing, and when lower-than-expected HIV prevalence is reported, as recently in Kenya and Zimbabwe, it can give the impression that things are 'not so bad' - when 7 to 10 percent of an adult population is living with HIV" [3]. While it may be that international opinion has become numbed by the persistence of the AIDS epidemic, it remains, to varying degrees in different regions, a prevalent and lasting feature of the global health landscape.

## Conclusions

The concept of AIDS exceptionalism developed as a Western response to an epidemic that threatened the lives and rights of specific populations in the developed world. As that epidemic was contained and effective treatment became available, the case for exceptionalism shifted to the international stage, where resources and organizations were mobilized to respond to the extreme need in developing countries. As a result, the numbers of PLHIV on treatment has increased each year. Infection rates in much of the world have stabilized.

These gains have been accompanied by criticisms of the type and size of response. It is argued that the HIV/AIDS response has done harm, as well as good, particularly by creating vertical programmes for a single disease, which may have diverted resources. As donor countries shift priorities, and in the context of the economic recession, the urgency around the HIV/AIDS response is once again declining.

This shift in policy and international priorities does not change the reality of an epidemic that, after three decades, is still unfolding. In southern Africa, the demographic effects of the generalized epidemic will shape societies for generations. In other parts of the world, HIV/AIDS continues to mark inequalities: one in 40 blacks, one in 10 men who have sex with men, and one in eight injection drug users in New York City are HIV positive [10].

Both the human rights approach, originally adopted by the HIV/AIDS response, and the more recent demands for universal access to treatment, remain relevant to the 33 million people living with HIV/AIDS and to their communities; these issues should also remain pertinent within global health policy. Meanwhile new challenges are developing, not the least of which is the need to successfully integrate the HIV/AIDS response within broader public health responses to the benefit of all. As Sachs notes in a commentary in *The Lancet*, "We are not overspending on AIDS but underspending on the rest ... The choice is not between AIDS, health systems, and other Millennium Development Goals. We can and must support them all" [42].

As how to best approach such challenges is debated, we must not lose sight of the approximate 2 million AIDS-related deaths that occur each year. Defining these deaths as either exceptional or unexceptional seems both callous and arbitrary.

## Competing interests

The authors declare that they have no competing interests.

## Authors' contributions

Both authors contributed to the research and writing of this paper, and read and approved the final manuscript.
